# Pollution by Antibiotics and Antimicrobial Resistance in LiveStock and Poultry Manure in China, and Countermeasures

**DOI:** 10.3390/antibiotics10050539

**Published:** 2021-05-06

**Authors:** Ming Tian, Xinmiao He, Yanzhong Feng, Wentao Wang, Heshu Chen, Ming Gong, Di Liu, Jihong Liu Clarke, André van Eerde

**Affiliations:** 1 Institute of Animal Husbandry, Heilongjiang Academy of Agricultural Sciences, Harbin 150086, China; tianming@haas.cn (M.T.); 1@haas.cn (X.H.); lixiang@haas.cn (Y.F.); 777@haas.cn (W.W.); chs18504348587@163.com (H.C.); 2 The Breeding Center of Felid of Hengdao He Zi (Heilong Jiang) China, Siberia Tiger Park Heilongjiang, Harbin 150086, China; littlebee6591208@163.com; 3 NIBIO, Norwegian Institute of Bioeconomy Research, P.O. Box 115, NO-1431 Ås, Norway; Andre.vanEerde@nibio.no

**Keywords:** antibiotics, antimicrobial resistance (AMR), bacteria, China, human and animal health, livestock and poultry manure

## Abstract

The demand for animal protein has increased considerably worldwide, especially in China, where large numbers of livestock and poultry are produced. Antibiotics have been widely applied to promote growth and prevent diseases. However, the overuse of antibiotics in animal feed has caused serious environmental and health risks, especially the wide spread of antimicrobial resistance (AMR), which seriously affects animal and human health, food safety, ecosystems, and the sustainable future development of animal protein production. Unfortunately, AMR has already become a worldwide challenge, so international cooperation is becoming more important for combatting it. China’s efforts and determination to restrict antibiotic usage through law enforcement and effective management are of significance. In this review, we address the pollution problems of antibiotics; in particular, the AMR in water, soil, and plants caused by livestock and poultry manure in China. The negative impact of widespread and intensive use of antibiotics in livestock production is discussed. To reduce and mitigate AMR problems, we emphasize in this review the development of antibiotic substitutes for the era of antibiotic prohibition.

## 1. Introduction

With the increasing demand for protein-rich food, global animal production has intensified considerably, especially in developing countries. Antibiotics are widely used in animal husbandry because of their functions in promoting growth and preventing disease. Antibiotics provide benefits, but also cause severe problems to food safety, human health, and the environment, leading to the antimicrobial resistance (AMR) problem [1]. It has been reported that by 2050, an estimated 10 million deaths per year worldwide will be attributable to AMR, and the economic burden in the health sector will reach as high as USD 100 trillion [2]. Given that AMR has become a big threat to global public health, it requires a joint effort and commitment by the international community, and across sectors, to mitigate and manage it.

It is reported that approximately 80% of all antibiotics sold in the United States are sold for use in animal agriculture to boost animal growth and protect them from infections [3]. Moreover, data for antimicrobial sales collected from 41 countries showed estimated global antimicrobial sales of 93,309 tonnes in 2017 and 104,079 tonnes in 2030, with a rise of 11.5% [4]. Hence, effective mitigation of the risks related to the extensive use of antimicrobials in animal farming and management of the AMR problem are essential, and require multi-disciplinary integrated approaches, such as the “One Health” approach. “One Health” involves a collaborative, multisectoral, and trans-disciplinary approach, operating at local, regional, national, and global levels, in order to achieve optimal health and well-being outcomes and emphasizing the interconnections between people, animals, plants, and their shared ecosystem and environment (http://www.who.int (accessed on 30 March 2021)). For instance, the recent outbreaks of swine influenza, Ebola, and the devastating ongoing Covid-19 pandemic are examples of zoonotic diseases transmitted between animals (including wildlife) and humans. Luckily, many countries have already taken action to reduce the application of antimicrobials in animal production; in particular China, the world’s largest meat consumer. Already in 2016, the Chinese government launched a national pilot program coupled with policy and regulations to decrease unnecessary antimicrobial use [4]. There is unfortunately no *quick fix* for the AMR problem, due to its complexity. It is clear that a ban on antibiotic feed additives will directly affect the disease resistance and normal growth cycle of livestock production, thus indirectly increasing the investment costs of livestock and poultry. Hence, there is an urgent need to tackle the AMR threat and find safe and sustainable solutions to ensure the animal protein food supply and protect human, as well as animal, health in the future. This review focuses on pollution by antibiotics and AMR in livestock and poultry manure and countermeasures against this in China, in light of China’s 14th 5-year plan, which was launched recently and recognized the increasing demand for animal protein food supply and security. 

### Distribution of Antibiotics and Antibiotic Resistance Genes in Animal Manure

The environmental pollution caused by antibiotics and their ecotoxicological effects has become a major global problem, especially the environmental pollution caused by the abuse of antibiotics in the livestock and poultry industry [5,6,7,8], which has become one of the current international research hotspots [9]. Studies have shown that only a small amount of the administered antibiotics participate in animal metabolism and are effectively used. Most antibiotics and their antimicrobial resistance genes induced in animals are directly discharged from the body with the urine and feces of animals [10,11,12], and the AMR genes carried by animal urine and feces are subsequently transmitted to environmental microorganisms [13]. Untreated feces containing residual antibiotics and/or AMR genes are often applied to farmland as organic fertilizer, which will exert pressure on the resistance of microorganisms in the soil environment, and induce further antibiotic resistance genes, causing serious environmental pollution, food safety challenges, and ecological toxicity [14]. AMR genes are frequently detected in livestock manure [15,16,17,18]. Among these genes, the most common types of resistance genes in livestock breeding are the tetracycline resistance gene and sulfonamide resistance genes [19,20]. Chee-Sanford et al. [21] found eight tetracycline resistance genes encoding resistance in septic tanks and groundwater samples near pig farms; these were tetracycline O (tet (O)), tetracycline Q (tet (Q)), tetracycline W (tet (W)), tetracycline M (tet (M)), tetracycline b P (tetb (P)), tetracycline S (tet (S)), tetracycline T (tet (T)), and oxytetracycline (otr (A)). This investigation showed that the unregulated discharge and treatment of livestock manure will cause serious AMR gene pollution in the surrounding water and soil environment.

China is a large agricultural country, and the livestock and poultry breeding industry has gradually changed from decentralized, to intensive, farming. The use of antibiotics is common, and the antibiotic-related pollution caused by livestock and poultry manure is prominent [22,23,24]. Animal manure has become a huge antibiotic resistance gene pool. Cheng et al. [25] confirmed the existence of the tetracycline resistance gene and sulfonamide resistance gene by studying livestock farms in eastern China; He et al. [26] measured the content of antibiotic resistance genes (ARGs) in the pig manure of three commercial pig farms in southern China, and found that 22 kinds of ARGs studied were detected in almost all samples, and the abundance was high. The detection rate of sulfonamide (sul), chloramphenicol (cml), and macrolidelincosamides–streptogramin B (MLSB) resistance genes was 100%. The antibiotic residues in animal feces found in recent years are summarized in Table 1. According to the data, tetracyclines (TCS), sulfonamides (SAS), and quinolones (QNS) were commonly residual in livestock manure, and the concentration of tetracycline antibiotics was high.

## 2. Distribution of Antibiotics and Antibiotic Resistance Genes in Water, Soil, and Plants

**Figure 1:**
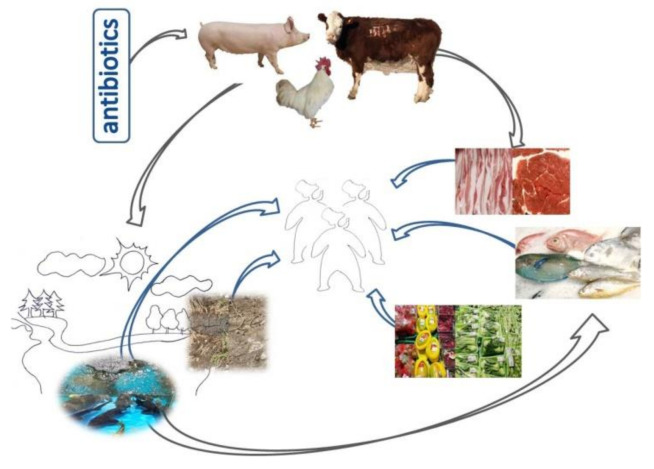
Veterinary antibiotics are widely used in the livestock and poultry production industry to accelerate the growth of livestock and poultry and treat diseases. This has led to the extensive spread of AMR and ARGs from animals to people, other animals, plants, the ecosystem, and the environment (soil and water pollution), as illustrated in this schematic figure.

It is estimated that 30–90% of antibiotics are excreted from the body, in the form of parent compounds or main metabolites, along with animal feces or urine [34]. High concentrations of antibiotics in livestock manure can enter the soil and water environment in various ways (Figure 1), thus causing pollution to the ecological environment. Residual antibiotics can enter the soil through animal manure and urine fertilization, and accumulate in the soil, affecting soil fertility, crop chlorophyll synthesis, enzyme secretion, and root growth. Antibiotic residues also affect soil microbial community structure and activity, and induce the generation and spread of antibiotic resistant microorganisms and resistance genes. The adsorption and degradation abilities of soil with regard to different kinds of antibiotics are different, with relative rates in the order tetracyclines > fluoroquinolones > macrolides > sulfonamides. After the manure applied to farmland is absorbed by soil, some antibiotics will enter into groundwater through infiltration or runoff with soil leaching, resulting in water pollution. In addition, the residual antibiotics in livestock and poultry feces will pollute the surface water with the wastewater discharged from livestock farms or with rainwater.

### 2.1. Distribution of Antibiotics and Antibiotic Resistance Genes (ARGs) in Water

Treated or untreated aquaculture wastewater is an important source of river pollution by antibiotics and ARGs. Xu et al. [35] monitored the occurrence of nine commonly used veterinary antibiotics in the Pearl River in flood and low water seasons, including antibiotics such as ofloxacin, norfloxacin, and amoxicillin. Only amoxicillin was not detected, while the other eight kinds of antibiotics were detected, with concentration ranges of 11–67 ng/L in the flood season and 66–460 ng/L in the low water season. Nine antibiotics, except doxycycline, were detected in the sewage from livestock farms in the north, middle, and south of Jiangsu Province and in the river water near the farms, with the highest concentration ranges of 0.44–169 μg/L and 0.46–4.66 μg/L [36]. In the Yellow River and its tributaries, the average concentrations of ofloxacin, norfloxacin, roxithromycin, erythromycin, and sulfamethoxazole were 25–152 ng/L, and 44–240 ng/L in some tributaries [37]. Li et al. [36] detected seventeen kinds of antibiotics, such as ofloxacin and enrofloxacin in Baiyangdian Lake water. In addition, antibiotics have been detected in groundwater in greenhouses, where animal manure is used as organic fertilizer to grow organic vegetables.

In addition to antibiotics, a large number of ARGs were detected in the river. For example, the detection rate of sulfonamide resistance genes (sul1 and sul2) in Tianjin Haihe River was 100%, and their abundance was high [38]; there were tet A and tet B2 tetracycline resistance genes in Pearl River water, and the detection rates were 43% and 40% [39], respectively. Jiang et al. [40] detected drug resistance genes in Huangpu River and a drinking water reservoir in Shanghai, and found two sulfonamide resistance genes, nine tetracycline resistance genes, and one β—lactam resistance gene. These water bodies polluted by drug-resistant bacteria are a potential threat to human beings, and direct drinking may cause harm to human health. 

The environmental risks of water pollution caused by antibiotics are now in focus and need the attention of the entire society. Antibiotics have been widely found in water, and are eventually absorbed or consumed by aquatic organisms. When antimicrobials are used in aquaculture seafood production, the AMR genes may enter into the food chain and eventually into the human body through seafood consumption. This could cause damage to human health over time and lead to drug resistance, which could have a great impact on the elderly, children, pregnant women, and other groups with weak immunity. Furthermore, because of the long-term enrichment of antimicrobials in water, some bacteria and fungi could acquire multi-drug resistance, and then form “superbugs”. Infections caused by superbugs are a true threat to human and animal health worldwide and can lead, not only to loss of life, but also to a heavy economic burden on the health sector (www.who.int (accessed on 30 March 2021)).

### 2.2. Distribution of Antibiotics and Antibiotic Resistance Genes in Soil

High concentrations of antibiotics can be detected in the soil around farms or after fertilizing with livestock manure. Ji et al. [27] studied the content of antibiotics in farmland soil near pig, cattle, and chicken farms in Shanghai and found the concentrations of tetracycline and oxytetracycline were 1.87–4.24 mg/kg dry matter (DM), while the concentrations of sulfadiazine, sulfamethoxazole, and sulfamethoxazole were 1.29–2.45 mg/kg DM. Hu et al. [11] investigated the content of antibiotics in the soil of organic vegetables planted with duck and pig manure in four different areas of Tianjin and found that 11 kinds of antibiotics (including tetracyclines, sulfonamides, and quinolones) were detected in the soil in winter, among which the content of oxytetracycline was the highest, reaching 2.68 mg/kg DM. Six kinds of antibiotics were detected in summer soil, among which the tetracycline content was the highest, reaching 2.5 μg/kg DM. River sediment is also often contaminated with antibiotics. Zhou et al. [41] investigated the contents of 17 kinds of antibiotics in the sediment of the Yellow River, Haihe River, and Liaohe River. The results showed that norfloxacin, enrofloxacin, ciprofloxacin, and oxytetracycline were among several antibiotics with high detection rates, and their contents reached 5770 ng/g DM, 1290 ng/g DM, 653 ng/g DM, and 652 ng/g DM, respectively.

The long-term abuse of antibiotics in the livestock and poultry breeding industry induces resistant strains in the intestinal tracts of breeding animals, and the discharge of a large amount of livestock and poultry manure will directly lead to non-point source pollution of ARGs [21]. According to Jensen et al. [42], animal manure fertilization leads to selective pressure on resistant bacteria in soil, and fertilization with animal manure is the main path for drug-resistant microorganisms and ARGs to enter the soil environment. Further studies have found that ARGs can, not only spread among strains in animal intestines, but also integrate into some mobile gene elements (such as plasmids, transposons, integrons, etc.) [43,44] and can also spread between indigenous soil bacteria and other microorganisms after entering the soil environment [45]. Under selective pressure, ARGs from pathogenic microorganisms spread among various microorganisms in the environment through gene horizontal transfer [46,47]. Moreover, heavy metals in animal feces (such as copper, zinc, and chromium) can also promote the horizontal transfer of drug-resistant genes [27]. Through this horizontal gene transfer, ARGs can migrate and transform in the soil, groundwater, and other environmental media. Owing to their strong adaptability, the indigenous microorganisms that obtain ARGs reproduce easily and become a reservoir of resistance genes [48].

### 2.3. Distribution of Antibiotics and Antibiotic Resistance Genes in Plants

Plants can absorb antibiotics from the soil where animal manure is applied. Hu et al. [8] detected the contents of 11 antibiotics in radish, rape, celery, and coriander planted with duck manure and pig manure as organic fertilizer in four greenhouses in four different regions of Tianjin. The results showed that ten kinds of antibiotics (0.1–57 μg/kg DM) were detected in radish, eight antibiotics (0.1–187 μg/kg DM) were detected in rape, seven antibiotics (0.1–20 μg/kg DM) were detected in celery, and seven antibiotics (0.1–532 μg/kg DM) were detected in coriander. In addition to common vegetables, antibiotics can also be detected in forage, corn, wheat, and peanut fertilized with animal manure [49]. Microorganisms in the soil can also be transferred to plants. These microorganisms transferred to plants live in the intercellular spaces or the cells of various tissues and organs of healthy plants at a certain stage, or all stages, and are called plant endophytes [50]. Many microorganisms in the soil carry drug resistance genes, which will indirectly carry resistance genes after they are transferred to plant tissues. Yang et al. [51] found that the endophytic bacteria of celery, Chinese cabbage, and cucumber planted with chicken manure generally had antibiotic resistance, and the resistance rate to cefalexin was the highest. Marti et al. [52] detected the resistance genes carried by endophytic bacteria of tomato, cucumber, pepper, radish, carrot, and lettuce planted with cow manure and pig manure. Compared with the control group, more types of ARG were detected in the group treated with animal manure. More importantly, through vegetables that can be eaten raw, ARGs in edible products and resistant plasmids in soil are likely to enter the human body along with food, thus increasing the antibiotic resistance of the human body [53]. Due to the special and hereditary nature of gene pollution, it is difficult to control and eliminate it. Once spread, it will cause long-term and irreversible harm to human health and ecosystems [54].

## 3. The Consequences of Antibiotic Abuse

### 3.1. Weaker Immune System in Livestock and Poultry

After a large number of antibiotics are ingested into the body, they will be distributed to the lymph nodes, kidney, liver, spleen, thymus, lung, bone, and other tissues and organs with blood circulation [55,56]. The immune capacity of animals will be gradually weakened, and the incidence of chronic diseases will increase. Antibiotics can also lead to the decrease of antigen quality, directly affecting the immune process, and thus have adverse effects on vaccination [57].

### 3.2. Cause Dysbacteriosis, Disease, or Secondary Infection in Livestock and Poultry

Although antibiotics have their own antibacterial spectrum, they not only inhibit pathogenic microorganisms, but also disturb the pattern of mutual restriction among populations or communities in the microbial flora. This leads to an imbalance of the microecology, resulting in the excessive reproduction of native bacteria or passing bacteria, further resulting in double infection or endogenous infection [58]. Especially, long-term and large-scale use of antibiotics will cause an imbalance of flora in the body; when the ecological balance is destroyed the harmful bacteria that lurk in the body take advantage of the opportunity to multiply and cause endogenous infection [59]. Antibiotics can eliminate the sensitive bacteria in the body, causing a large number of vacancies on some microbial attachment points in the body, providing opportunities for external drug-resistant bacteria to enter, thus causing exogenous infection [60].

### 3.3. Residues in Animal Products and the Environment

Drug residue is one of the controversies regarding adding antibiotics to feed. After being absorbed into the body, antibiotics are distributed to almost all organs of the body, especially the liver. Roughly 60–85% of antibiotics are excreted in different forms, such as feces or bile. Some antibiotics with stable properties can remain stable for a long time after being excreted into the environment, resulting in drug residues in the environment. These residual drugs are slowly accumulated in the human body and plants through animal products and the environment, and finally gathered in the human body through various pathways, resulting in the production of a large number of drug-resistant strains, loss of resistance to certain diseases, or toxic effects on the body due to a large accumulation [7].

### 3.4. Antibiotic Resistance of Pathogens

The formation of antibiotic-resistant pathogens is a global problem. At present, there is almost no antibiotic without antibiotic resistant pathogens. In particular, the long-term use of antibiotics in animals at doses lower than the treatment dose (such as preventive doses and growth promoting doses) can accelerate the production of antibiotic-resistant bacteria. Once antibiotic-resistant bacteria are produced, they can spread among animals, which will make animals in large-scale breeding facilities become a huge reservoir of ARGs. The antibiotic resistance of antibiotic-resistant bacteria from animals can be transferred to human beings, which has a great impact on human health. According to results published in the Proceedings of the National Academy of Sciences, 149 unique ARGs have been found in Chinese commercial pig farms, and the levels of some ARGs are 192 to 28,000 times higher than those of the control samples [4].

### 3.5. Negative Impact of Antibiotics and ARGs on Human Health

Previous studies have shown that penicillin, streptomycin, sulfonamides, and other antibiotics cause allergies; chloramphenicol causes regenerative, dysfunctional and hemolytic anemia, thrombocytopenia, and liver injury; tetracyclines cause photosensitivity and gastrointestinal reaction; olaquindox is a gene inducer; furazolidone induces animal carcinogenesis; aminoglycoside antibiotics cause nephrotoxicity; and fluoroquinolone keto antibiotics can cause mitochondrial damage [61,62].

The harm caused by ARGs is mainly manifested in the resistance of bacteria to antibiotics, which makes these ineffective or reduces their effect. At present, infections caused by antibiotic-resistant bacteria are becoming more common around the world [63,64]. According to a survey of infectious disease experts conducted by the emerging infection network of the United States, more than 60% of the participants said that they had seen extensive antibiotic-resistant and incurable bacterial infections in the past year [65]. The World Health Organization warned in 2014 that the antibiotic resistance of bacteria had become a terrible crisis [66]. It has been reported that by 2050, the global bacterial infectious diseases caused by antibiotic resistance are expected to cause 10 million deaths per year, 1.8 million more than those due to cancer [67].

## 4. Prohibition of Antibiotics in the World

In the 1950s, the United States Food and Drug Administration (FDA) approved, for the first time, the addition of antibiotics to feed, and countries around the world began to study the application of antibiotic additives; since the 1960s, countries have begun to use a large number of antibiotic additives in feed. With the development of antibiotic resistance and the intensive use of antibiotics, people have paid more and more attention to the potential harm of antibiotics. Since the 1980s, based on food safety considerations, countries have enforced strict restrictions on the use of antibiotic additives. In 1986, Sweden completely banned the use of antibiotics in animal feed. In January 2006, EU countries completely banned the addition of any antibiotics to feed. In 2010, the U.S. Food and Drug Administration began to call for a gradual ban on the use of “antibiotics with important medical uses” in animal husbandry. The latest revision of the Drug Law of Germany introduced the concept of minimum antibiotic use. Antibiotic monitoring involves 95% of poultry farms and about 90% of pig farms in Germany. In September 2015, the European Union published guidelines on the prudent use of antimicrobial veterinary drugs. The use of antibiotics as feed growth promoters was banned in South Korea in 2011.

China attaches great importance to the management of antibiotics. In recent years, with the improved understanding of the hazards of antibiotics, the Ministry of Agriculture of China promulgated a series of announcements, such as the No. 168 notice “the use standard of feed drug additives” which was promulgated in 2001, and announcement No. 193 “list of prohibited veterinary drugs and other compounds in food animals”, which was promulgated in 2002, standardizing the use of antibiotic feed additives. At the end of 2015, the use of lomefloxacin, pefloxacin, ofloxacin, and norfloxacin was prohibited in food animals. In November 2016, regulations were issued to stop the use of colistin sulfate as an feed additive. On 20 April 2018, the state issued a pilot work plan for the reduction of veterinary antimicrobial use (2018–2021). On 10 July 2019, the No. 194 announcement of the Ministry of Agriculture and Rural Areas stipulated that, from 1 January 2020, all kinds of growth promoting drug feed additives, except traditional Chinese medicines, should be withdrawn, veterinary drug manufacturers should stop production, and the importers of veterinary drug agents should stop importing the corresponding veterinary drug products, and at the same time they cancelled the corresponding veterinary drug product approval number and import veterinary drug registration certificate. This marks the withdrawal of 12 growth promoting drug feed additives from the historical stage, and China has officially entered the era of it being “forbidden to add antibiotics to feed”.

## 5. Development of Antibiotic Substitutes

In recent years, AMR has become a global threat to human and animal health, as well as to the ecosystem and environment. In order to reduce and eventually eliminate the AMR problem in the future, antibiotic substitutes play an important role. More and more antibiotic substitutes have been used to reduce or even replace the use of antibiotics in livestock production (Table 2).

### 5.1. Plant Bioactive Substances

#### 5.1.1. Essential Oils

At present, there are about 4500 kinds of essential oils and other bioactive components extracted from the flowers, seeds, leaves, bark, and fruits of various plants. The main components of essential oils include carvacrol, citronellol, geraniol, eugenol, and thymol [68]. Some essential oils have antibacterial, antifungal, antiviral, and anti-inflammatory properties because their components can interact with bacterial membrane lipids and cause cell lysis. For example, terpinene-4-ol, α—Terpinene, and 8-cineole in tea tree oil can cause autolysis of *Staphylococcus aureus* and promote the formation of intermediates [69]. In addition to inhibiting pathogens, essential oils also have the potential to improve the growth performance of livestock and poultry by increasing feed palatability [70]. However, their in vivo antibacterial activity is generally not as obvious as that in vitro, due to their easy inactivation and poor absorption in vivo [71,72]. Some essential oils have adverse effects on livestock and poultry [73,74]. Generally, these additives have shown good application prospects in laboratory research, but they often fail to achieve ideal results in livestock and poultry diets.

#### 5.1.2. Condensed Tannins (CT)

CT is a kind of oligomeric or polymerized flavonoid, which is composed of flavane-3-ol. It mainly includes catechin, epicatechin, gallocatechin, and epigallocatechin. CT has antibacterial, antiparasitic, antioxidant, and immunomodulatory activities. CT can be used as a natural substitute for antibiotics in feed [75]. The effects of feeding diets containing CT, or directly supplementing CT, on intestinal health and production performance of livestock and poultry are quite variable. The inconsistent effect of adding CT is related to the changes of chemical composition and concentration in various plants and plant tissues [76]. Another reason is that the biological activity changes during extraction and is similar to other components in the diet during storage and feeding. The interaction produces antinutritional properties, reducing the digestibility of nutrients [77] and the antibacterial activity of CT in the digestive tract [78]. These effects are more pronounced in ruminants and poultry than in pigs.

### 5.2. Phages

Phages are predators of bacteria, destroying half of the world’s bacteria every 48 h [79]. As early as 1917, phages were used to treat human infections. Most of the known phages can only bind specifically to the bacteria expressing the phage tail fiber recognition site. In order to increase the host range and prevent the bacteria from producing resistance to the phage, the phages used for treatment are usually a mixture of multiple phages. Although phages have been studied in detail, it is still difficult to find phages suitable for animal husbandry. At present, only phages with an anti-hemolytic activity for *Mannheimia* have been isolated [80,81]. Phages have the potential to control pathogens, especially those that have shown antibiotic resistance in livestock and poultry. However, the phages used to treat infection must be easy to cultivate and reproduce, and easy to store without inactivation; moreover, the search for phages suitable for the treatment of animal diseases is still in the initial stages, and further research is needed to verify their potential efficacy.

### 5.3. Vaccines

Vaccines are widely used to prevent bacterial and viral infections in livestock and poultry, and are currently the most promising alternative to antibiotics. Differently from the therapeutic effect of antibiotics, the most important value of vaccine is prevention, which can not only prevent virus infection but also prevent bacterial infection. Conventional vaccines usually use heat inactivated bacteria or viruses, or active modified and purified components. However, there is a direct effect of vaccination on the clinical status of the host, and therefore, there is a need for vaccination before the clinical response. It is very difficult to develop vaccines against intestinal microorganisms, and only a few vaccines can maintain their efficacy against pathogens in the intestinal environment. At the same time, in order to control the disease, it is necessary to realize whole group vaccination, otherwise the infectious pathogens can continue to spread among the unvaccinated individuals.

### 5.4. Probiotics

Probiotics can inhibit pathogens, change metabolism, and regulate immunity [82]. Probiotics can affect pathogens and inhibit bacterial growth by producing bacteriocin (antimicrobial peptide) [83], organic acids, peroxide [84], or competitive nutrients [85]. The symbiotic flora was changed by interaction with the microbial flora in vivo, the incidence of diarrhea was reduced [86], and feed efficiency was improved [87,88]. Probiotics help to promote the integrity of the intestinal barrier by inducing anti-inflammatory cytokines to reduce intestinal inflammation [89]. Non-specific and specific immune responses were regulated by the interaction with intestinal associated lymphocytes [90]. Most probiotics are isolated from the gut or fermented food, and can be commercialized mainly based on their high-yield and non-volatile performance, while the probiotics on, or in, the organism are ranked in the secondary position [91]. Generally, probiotics used in early stage livestock and poultry are basically grafted from human probiotics, so they may not be suitable for livestock or poultry. Currently, people are trying to isolate and cultivate probiotics more suitable for livestock and poultry.

### 5.5. Antimicrobial Peptides

An antimicrobial peptide (AMP) is a kind of polypeptide with a strong antibacterial effect. Antibacterial peptides have the advantages of a broad antibacterial spectrum, immune function, promoting growth, and not easily producing antibiotic resistance. They have a broad application potential in animal husbandry production. Research efforts have been made to identify and characterize AMPs in different organisms, and a large number of AMPs have been reported, even from organisms such as the Arctic cold-water red king crab [92]. The AMPs identified and characterized from the red king crab are being further investigated to reveal their functions. 

### 5.6. Acidifiers

Acidifiers can be divided into organic, inorganic, and compound acidifiers, which can improve the acidity value and enzyme activity of the diet and promote the absorption of nutrients. It was found that adding different levels of acidifier in sow feed can improve sow feed intake and promote piglet growth. Organic acids can reduce the infection rate and spread of Salmonella in pigs. The results of a Limin test showed that adding 1250 mg/kg metabolic organic acids to the diet could promote protein deposition in growing finishing pigs, thus improving their growth performance.

### 5.7. Oligosaccharides

Oligosaccharide is the general term for low molecular weight sugars with a straight or branched chain formed by two or more monosaccharides (generally 2–10) linked by a glycosidic bond. They have physical and chemical properties such as high temperature resistance, stability, and non-toxicity. At present, functional oligosaccharides are mainly used as feed additives. They can improve the microbial community structure of the digestive tract, improve the immunity of the body, promote the development of the digestive tract, and enhance the digestion and absorption capacity of animals. Dietary chitosan can promote the growth of piglets, improve the intestinal barrier function, and increase the quantity of bifidobacteria and lactic acid bacteria [93]. Hasunuma et al. [94] found that the daily gain and feed conversion efficiency of Holstein calves could be significantly improved by feeding them fiber oligosaccharides in their diet.

With the restriction of the use of feed antibiotics, there is an opportunity for enterprises and institutions to actively search for technical means and products that can effectively reduce or even replace these. However, the antibacterial efficacy of these substances has limitations. Compared with antibiotics, the mechanisms by which these substitutes inhibit or kill bacteria are still unclear, and their mode of action needs to be further understood.

## 6. Countermeasures of the Chinese Breeding Industry in the Era of Banning Antibiotics

AMR has become a global threat to human and animal health in addition to the negative impact on the ecosystem and environment. Stakeholders’ involvement is essential in order to mitigate the AMR problem. China has made efforts to control AMR spreading by policy enforcement, as well as the participation of the industry and livestock farmers in identifying and using alternative additives replacing antibiotics, as well as effective prevention, management strategy, and diagnosis through the support of veterinary and training courses.

There is a need for farmers to realize that the long-term use of antibiotics or abuse of antibiotics will bring irreversible harm to human health and the living environment. The government can regularly organize training on non-resistance breeding; vigorously publicize the harm of antibiotic abuse and the benefits of breeding without antibiotic overuse to large numbers of farmers, changing the farmers’ perception; hire experts to train the framers in breeding, so as to improve their professional skills; establish a non-resistant breeding pilot; and promote non-resistant breeding technology.

### 6.1. Effective Immunization and Improvement of the Sanitation of the Internal and Surrounding Environment of Livestock Facilities

In the past, environmental hygiene was not considered in agriculture, resulting in poor environmental sanitation, which led to the large-scale infection of livestock. Farms (households) could only use antibiotics to treat them. Therefore, it is necessary to establish ecological breeding areas. At the same time, other related industries should be allocated according to local conditions to form an efficient and pollution-free support engineering system. The development of resources and the ecological balance should be organically combined to reduce the occurrence of livestock diseases. According to a pre-established immunization plan, we should improve the vaccination of livestock and reduce the incidence rate of livestock and poultry diseases [95]. Regular cleaning and disinfection inside and outside the livestock housing can create a good feeding environment, which can also make the livestock and poultry healthy and reduce the mortality rate [96]. 

### 6.2. Use Alternatives to Antibiotics

Controlling pathogens from the source, improving the health supervision system and biosafety route, and selecting some mature antibiotic substitutes (such as Chinese herbal medicine preparations, enzyme preparations, microbial preparations, etc.) can improve the economic benefits of agriculture [97,98,99]. 

## 7. Outlook and Conclusions

China is a large country for livestock and aquaculture production, and also a significant country for the production and use of veterinary antibiotics. It is an undeniable fact that veterinary antibiotics play an important role in animal husbandry production, the prevention and control of animal diseases, the improvement of breeding production efficiency, and guaranteeing an effective supply of livestock and poultry products. However, for a long time, the irrational use of veterinary antibiotics in Chinese animal husbandry production has led to an increase in the antibiotic resistance rate of animal derived bacteria and the emergence of "super-bacteria"; the residues of veterinary drugs and pesticides in animals aggravate the toxic and side effects of veterinary antibacterial drugs. The residual drugs are discharged into the outside world, polluting the soil and water sources, resulting in the pollution of the ecological environment. This threatens the safety of livestock and aquatic product production and quality, public health, and ecological environment security. Therefore, China has taken positive and decisive measures to ban the use of growth promoting feed drug additives. This is of great practical significance for the transformation and upgrading of Chinese animal husbandry, ensuring the safety of the people’s food and the export trade in livestock and poultry products to the world. 

In order to tackle the AMR problem, public awareness of the AMR threat, public engagement in mitigating AMR spread, and both basic and advanced medical education and training on the causes of the AMR problem, as well as solutions to reduce and control AM, are essential, and will play a pivotal role in the effective management of AMR globally [100]. We should also pay attention to the control of antibiotic pollution from the perspective of public health. We should control antibiotic pollution from the source, establish regular monitoring and linkage mechanisms between the upstream and downstream, and realize a dynamic assessment of antibiotic risk. At the same time, in ecological environment management, regulatory departments need to monitor the sources, uses, and disposal of antibiotics, so as to improve the efficiency of the linkage mechanism. It is necessary to improve the removal levels of sewage and wastewater treatment equipment, increase the investment in prevention and control funds, accelerate the research and development of new materials and technologies, and pay attention to education and the popularization of knowledge on antibiotics [100].

The use of antibiotics and antibiotic resistance is a worldwide challenge, and the research and development of antibiotic substitutes needs to continue. Therefore, international cooperation is becoming more and more important. China needs to work with other countries to cope with the challenges of the post-antibiotic era and develop new antibiotics or antibiotic substitutes, while strictly controlling the use of antibiotics. It is believed that with the joint efforts of the international community, the effective management and sustainable use of antibiotics can be realized.

## Figures and Tables

**Table 1:** Residues of antibiotics in animal manure.

Fecal Type	Region	Antibiotic Residues (μg/kg)		
		Tetracyclines	Sulfonamides	Quinolones
Pig	Tianjin	0.08–183.5	0.1–32.5	0.1–24.7
	Shandong	0.15–59.1	0.04–4.1	0.08–44.15
	Shanghai	12.27–18.70	4.88–7.56	ND
	Beijing	3.33–12.3	0.17–1.06	0.41–1.45
	Jiangsu	0.51–0.56	0.01–0.43	ND
	Sichuan	0.015–215.3	0.002–6.79	0.012–0.125
	Three northeastern provinces	0.32–56.81	0.1–4.84	0.14–3.18
Cattle	Shandong	0.24–59.06	0.06–0.36	0.41–46.7
	Shanghai	12.01–21.36	4.57–9.36	ND
	Jiangsu	0.52	ND	ND
	Sichuan	0.015–2.5	0.002–0.07	0.01–0.74
	Three northeastern provinces	0.21–10.37	0.08–1.02	0.61–4.17
Chicken	Shandong	0.14–17.68	0.02–6.04	ND
	Tianjin	0.6–173.2	0.3–26.4	0.3–21.9
	Jiangsu	8.9–65.7	0.75–2.18	8.73
	Sichuan	0.014–416.8	0.002–2.1	0.01–8.58
	Three northeastern provinces	0.54–13.39	0.09–7.11	0.13–15.43

Data are from [11,27,28,29,30,31,32,33]; ND = not detected.

**Table 2:** Classification of Antibiotic Substitutes.

Category	Plant Active Substances		Phages	Vaccines	Probiotics	Antimicrobial Peptides	Acidifiers	Oligosaccharides
Classification composition	Essential oil	Condensed tannins	-	Heat inactivated bacteria, viruses, active modified and purified components	Lactic acid bacteria, yeast, Lactobacillus, etc.	Polypeptides	Organic, inorganic and compound acidifiers	Soybean oligosaccharides, fructooligosaccharides, xylooligosaccharides, chitooligosaccharides, mannan oligosaccharides, and galactooligosaccharides
	Carvacrol, citronellol, geraniol, eugenol, thymol	Oligomeric or polymerized flavonoids consisting of flavane-3-ol. Mainly includes catechin, epicatechin, gallocatechin, epigallocatechin						
Function	Antibacterial, antifungal, anti-virus, anti-inflammatory, increase feed palatability	Antibacterial, antiparasitic, antioxidant, immunomodulatory activities	Predate on bacteria	Prevention of virus and bacterial infection	Inhibit pathogens, change microbial metabolism and regulate immunity	Broad antibacterial spectrum, immune function, promoting growth, not easy to produce drug resistance	Improve the acidity value and enzyme activity, promote the absorption of nutrients	Improve the microbial community structure of the digestive tract, improve the immunity of the organism, promote the development of the digestive tract, and enhance the digestion and absorption of animals
Defects	Easy to be inactivated in vivo and has no obvious antibacterial activity	The biological activity is easy to change, and the antinutritional property is produced by nonselective binding	Phages suitable for animal husbandry have not been found	Difficult to develop a vaccine against intestinal microorganisms	Unstable probiotics	-	-	-

“-” means information not found.
